# Effects of Graphene Reinforcement on Static Bending, Free Vibration, and Torsion of Wind Turbine Blades

**DOI:** 10.3390/ma17133332

**Published:** 2024-07-05

**Authors:** Hyeong Jin Kim, Jin-Rae Cho

**Affiliations:** 1 Department of Mechanical Engineering, University College London, London WC1E 7JE, UK; hj.kim.22@ucl.ac.uk; 2 Department of Naval Architecture and Ocean Engineering, Hongik University, Sejong 30016, Republic of Korea

**Keywords:** wind turbine blades, graphene reinforcement, graphene platelet-reinforced composites (GPLRC), mechanical characteristics, finite element structural analysis

## Abstract

Renewable energy markets, particularly wind energy, have experienced remarkable growth, predominantly driven by the urgent need for decarbonization in the face of accelerating global warming. As the wind energy sector expands and turbines increase in size, there is a growing demand for advanced composite materials that offer both high strength and low density. Among these materials, graphene stands out for its excellent mechanical properties and low density. Incorporating graphene reinforcement into wind turbine blades has the potential to enhance generation efficiency and reduce the construction costs of foundation structures. As a pilot study of graphene reinforcement on wind turbine blades, this study aims to investigate the variations of mechanical characteristics and weights between traditional fiberglass-based blades and those reinforced with graphene platelets (GPLs). A finite element model of the SNL 61.5 m horizontal wind turbine blade is used and validated by comparing the analysis results with those presented in the existing literature. Case studies are conducted to explore the effects of graphene reinforcement on wind turbine blades in terms of mechanical characteristics, such as free vibration, bending, and torsional deformation. Furthermore, the masses and fabrication costs are compared among fiberglass, CNTRC, and GPLRC-based wind turbine blades. Finally, the results obtained from this study demonstrate the effectiveness of graphene reinforcement on wind turbine blades in terms of both their mechanical performance and weight reduction.

## 1. Introduction

Abnormal weather conditions have occurred worldwide due to the use of fossil fuels over the past centuries and the resulting surge in carbon emissions. International efforts have been made to prevent such climate change, including the Paris Agreement [1] and the International Maritime Organization (IMO)’s regulations on greenhouse gas emissions [2]. As alternatives to existing fossil fuels, renewable energy sources that are sustainable with less environmental destruction, such as wind energy, solar energy, and hydroelectric energy, have garnered attention. Among them, wind energy is expected to represent over 30% of the world’s electricity generation by 2050 [3].

With the recent expansion of the wind energy market, the size of wind turbines has gradually increased, including the development of ultra-large wind turbines of more than 15 MW. In addition, research is being actively conducted to increase power generation efficiency and decrease initial construction costs. Most of all, wind turbine blades are key structures that directly affect power generation efficiency and construction costs. Fiberglass composites are being used as the primary material for wind turbine blades, but research cases on the application of new materials such as basalt–carbon hybrid fibers, SiO_2_ and Al_2_O_3_, bamboo, and carbon fibers have been reported of late.

Mengal et al. [4] presented a comparison of material properties between glass and carbon fibers used for wind blades. They reported that the traditional glass and carbon fibers can be partially replaced with basalt fibers, improving economic efficiency. Chikhradze et al. [5] compared the mechanical properties of hybrid composites using basalt, glass, and carbon fibers, and reported that expensive carbon fibers can be partially replaced with basalt fibers. Ong and Tsai [6] examined the economic efficiency according to the proportion of carbon fibers reinforced into hybrid composites. Holmes et al. [7,8] reported that bamboo-epoxy laminated composites have sufficient strength and stiffness to replace conventional glass fiber composites for the use of wind turbine blades. Shen-xue et al. [9] found from their experimental study that bamboo materials have sufficient strength for wind turbine blades. Ennis et al. [10] assessed the commercial viability of developing cost-competitive carbon fiber composites suited for wind turbine blades. Paquette et al. [11] demonstrated the use of carbon fiber in subscale blades and investigated advanced structural concepts through the blade system design study.

While many studies have been conducted on the development and application of new materials, academia and various industries have paid attention to graphene platelets (GPLs) as the nanofiller for composite reinforcement. GPLs, known as ultra-light and high-strength nanomaterials, have similar material properties to CNTs, but their production and sales costs are lower. Moreover, GPLs have a significantly larger surface area than CNTs, enabling more flexible interaction and load transfer within the matrix [12]. For this reason, GPL-reinforced composites (GPLRC) have attracted more attention than CNT-reinforced composites (CNTRC) over the last ten years [13], and numerous research cases can be found in the literature. Notably, Rafiee et al. [14] theoretically and experimentally proved the superiority of the epoxy composite reinforced with 0.1 wt.% of GPLs in terms of mechanical properties (e.g., strength, stiffness, and fracture toughness) to pure epoxy materials as well as single-walled CNTs (SWCNTs) and multi-walled CNTs (MWCNTs) with the same weight fraction. Rafiee et al. [15] proved that reinforcement with 0.1 wt.% of GPLs increased the buckling strength of the beam by 51.5% compared to pure epoxy materials, and the critical buckling strength by 42.8 and 31.8% compared to the cases reinforced with the same weight of SWCNTs and MWCNTs, respectively. In addition, various research cases on GPL reinforcement have been summarized systematically in the existing literature [16,17,18].

As mentioned earlier, nanomaterials, such as GPLs, have been considered promising future materials because they have a large reinforcement effect even in small amounts; however, the industrial use of most nanomaterials (e.g., CNTs) is limited because mass production methods at low cost have not been developed yet. GPLs, however, can be utilized in various industries because their mass production is possible at relatively low cost [19]. The actual cases that applied GPLs to metals, concrete, electronic equipment, and sensors can be found in the literature [20,21,22]. Although various structures that apply GPLs have been developed, there is still no reported case on the application of GPLs to wind turbine blades.

In this context, the effects of the application of GPLs on the mechanical characteristics of wind turbine blades, such as natural frequency, bending, and torsion, are closely investigated, and the applicability of GPLs as future materials is examined in this study. In this study, to obtain more reliable and realistic results, the finite element model was created by referring to the SNL 61.5 m model, which is a 5 MW-class wind turbine blade [23], and the aerodynamic loads acting on the blade were calculated at the rated wind speed based on the blade element momentum theory (BEMT). Consequently, the mechanical characteristics of the wind turbine blade according to the volume fraction of GPL were analyzed in detail through numerical analysis based on the finite element method, and the blade was compared with those composed of existing fiberglass composites to examine the superiority of GPL-reinforced wind turbine blades.

## 2. Finite Element Modeling of GPL-Reinforced Wind Turbine Blade

### 2.1. Material Modeling of GPLRC

In this study, effective material properties were calculated by mixing the material properties of GPLs (nanofiller) and epoxy (matrix) to model GPLRC (nanocomposite). The interfaces between the matrix and GPLs are assumed to be perfectly bonded. Table 1 lists the material properties of these materials. The effective material properties of Poisson’s ratio ν and the density ρ are calculated using Equations (1) and (2) based on the linear rule of mixture.

(1) $$\nu_{eff}=V_{GPL}\;\nu_{GPL}+V_{m}\;\nu_{m}$$

(2) $$\rho_{eff}=V_{GPL}\;\rho_{GPL}+V_{m}\;\rho_{m}$$

where V is the volume fraction of the material. Subscripts eff, GPL, and m represent the effective material property, GPL, and matrix (epoxy).

The effective elastic modulus of GPLRC, $E_{eff}$, was modeled using the Halpin–Tsai micromechanical modeling technique presented in Equation (3), where L, T, GPL, and m represent the longitudinal direction, transverse direction, graphene platelet, and matrix.

(3) $$E_{eff}=\frac{3}{8}\cdot \frac{1+\xi_{L}\eta_{L}V_{GPL}}{1-\eta_{L}V_{GPL}}E_{m}+\frac{5}{8}\cdot \frac{1+\xi_{T}\eta_{T}V_{GPL}}{1-\eta_{T}V_{GPL}}E_{m}$$

where $E_{m}$ and $V_{GPL}$ indicate the elastic modulus of the matrix and the GPL volume fraction, respectively. Two parameters, $\eta_{L}$ and $\eta_{T}$, are defined by

(4) $$\eta_{L}=\frac{E_{GPL}-E_{m}}{E_{GPL}+\xi_{L}E_{m}},\;\eta_{T}=\frac{E_{GPL}-E_{m}}{E_{GPL}+\xi_{T}E_{m}}$$

with the geometry parameters given by

(5) $$\xi_{L}=\frac{2l_{GPL}}{t_{GPL}},\;\xi_{T}=\frac{2w_{GPL}}{t_{GPL}}$$

Here, the length $l_{GPL}$, width $w_{GPL}$, and thickness $t_{GPL}$ of GPLs were set to $l_{GPL}=2.5\;\mu m$, $w_{GPL}=1.5\;\mu m$, and $t_{GPL}=1.5\;\mu m$, according to the values presented in a study by Rafiee et al. [14]. Table 2 shows the calculated effective material properties of GPLRC as examples of the material modeling technique introduced above.

### 2.2. Geometry and Composite Layup of Wind Turbine Blade

**Figure 1:**
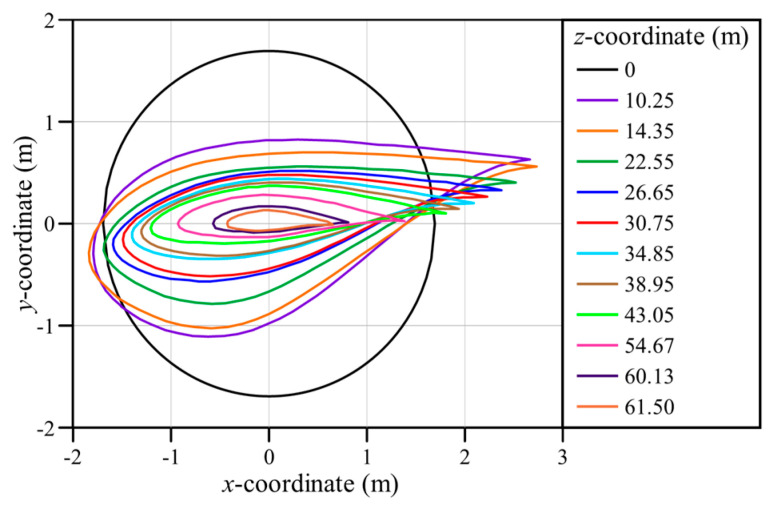
Airfoil distribution of the SNL 61.5 m wind turbine blade.

The main materials that constitute wind turbine blades these days are fiberglass composites. It is necessary first to identify the characteristics of existing fiberglass composite-based blades before analyzing the changes in the mechanical characteristics of wind turbine blades caused by GPL reinforcement. Therefore, a finite element model for static bending and twisting and free vibration of a fiberglass composite-based blade was created in this study by referring to the SNL 61.5 m model, a 5 MW-class wind turbine blade. As the name of the target model suggests, the blade span length is 61.5 m. Table A1 shows the parameters required to create the blade geometry, including the airfoil type, chord length, and aerodynamic center. In Table A1, the twist angle represents the initial twist angle of each airfoil cross-section, as shown in Figure 1. Other detailed geometric information for the target model is included in the report by Resor [23].

**Figure 2:**
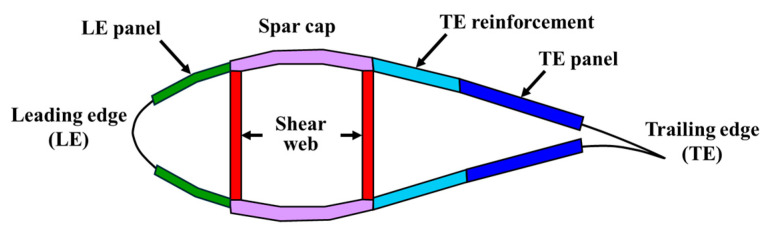
Cross-section view of wind turbine blade.

As shown in Figure 2, the cross-section of the wind turbine blade consists of structures, such as the leading edge (LE), LE panel, spar cap, trailing edge (TE), TE reinforcement, and TE panel. The materials used and layup vary depending on each structure and the position in the span direction. Gelcoat, E-LT-5500 (UD), Saertex (DB), SNL (Triax), Foam, and Carbon (UD) materials are used in the composite laminate of the SNL 61.5 m blade model. The material properties of each material are listed in Table 3. Here, E-LT-5500 (UD) and Saertex (DB) are composed of uni-axial fiberglass and double-bias fiberglass, respectively, while SNL (Triax) is a material that uses both [23].

**Figure 3:**
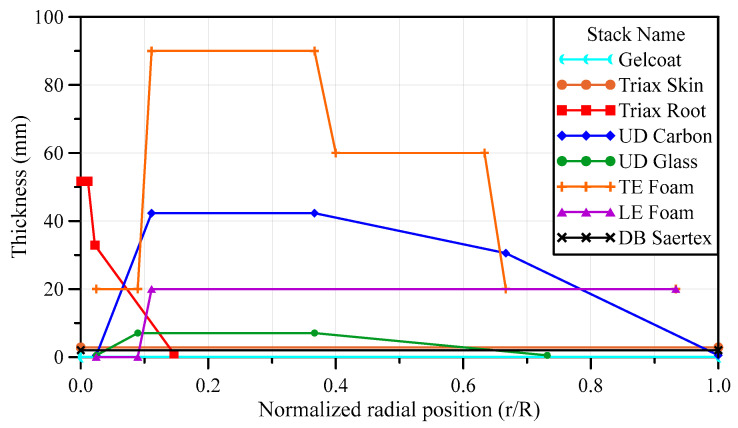
Thickness-wise material distributions of SNL 61.5 m wind turbine blade along the blade span.

**Figure 4:**
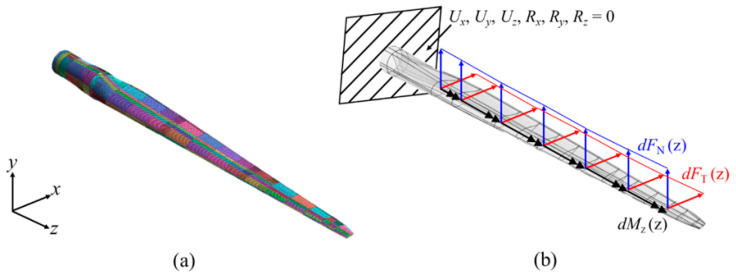
The SNL 61.5 m blade: (**a**) finite element model, (**b**) boundary and loading conditions.

As aforementioned, the material and thickness of the composite laminate vary depending on the cross-sectional structure of the blade and the position in the span direction. Table A2 and Table A3 list the stack IDs, names, and stacking sequences of composites, whereas Figure 3 shows the thickness distribution of each stack, where R and r denote the blade length and the position in the blade span direction, which is consistently used hereafter. In this study, the target model was created using midas-NFX, a commercial finite element analysis program, and the composite laminate model was created using the composite shell element. The element size was determined to be 80 mm × 80 mm by referring to the finite element model in a report by Resor [23]. Figure 4 shows the finite element model created for numerical analysis in this study, in addition to boundary and loading conditions.

### 2.3. Aerodynamic Loads Acting on Wind Turbine Blade

During the operation of a wind turbine, various loads, such as aerodynamic loads, inertial loads, and gravitational loads, act on the blade. In particular, it is known that large-deflection bending and torsional deformation of the blade are mainly caused by aerodynamic loads. Therefore, it is necessary to conduct finite element analysis by reflecting similar aerodynamic loads to reality to analyze the mechanical characteristics of the blade precisely. Computational fluid dynamics (CFD) and BEMT have been mainly used to calculate the aerodynamic loads applied to wind turbine blades [25]. Since CFD is costly and requires considerable modeling work and analysis time, BEMT-based aerodynamic load calculation methods have been used widely.

**Figure 5:**
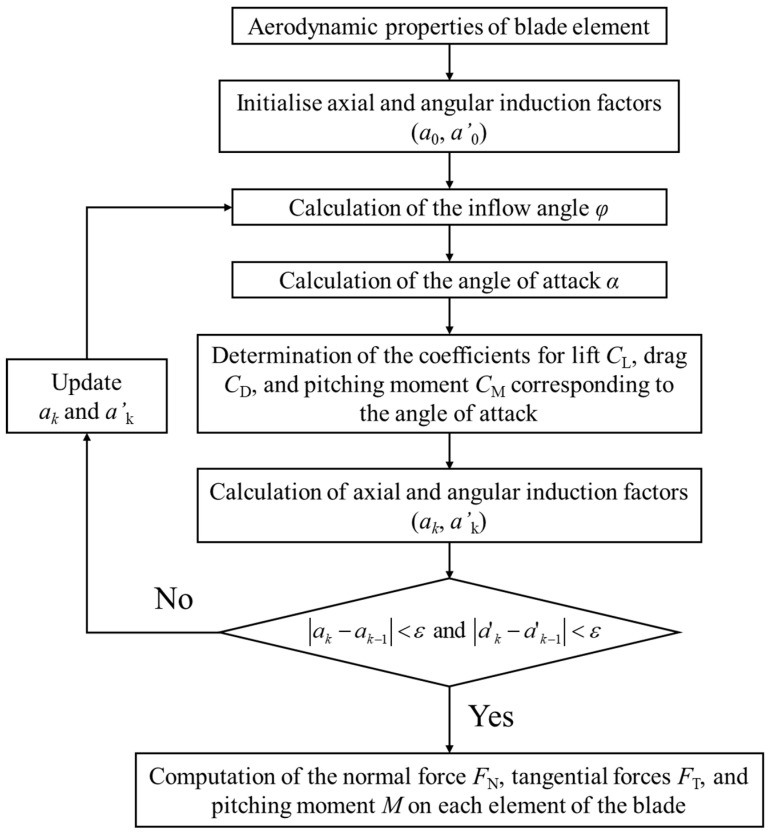
Procedures for calculating aerodynamic loads acting on wind turbine blades.

In this study, aerodynamic loads acting on the blade were calculated based on BEMT and then reflected in finite element analysis. Figure 5 shows the process of calculating aerodynamic loads using BEMT. Here, zero is used as the initial values of the axial and angular induction indices $a_{0}$ and $a\prime_{0}$, and the inflow angle φ is calculated through the following equation.

(6) $$\tan\varphi =\frac{(1-a)V_{0}}{(1+a\prime )\Omega r}$$

where $V_{0}$ is the free-stream velocity (wind speed in this paper). r and Ω are the position in the blade span direction and the angular velocity of the rotor, respectively. In this study, the rated wind speed (11.4 m/s) and rated rotor speed (12.1 rpm) of the 5 MW-class blade model presented in the NREL report were used [26].

**Figure 6:**
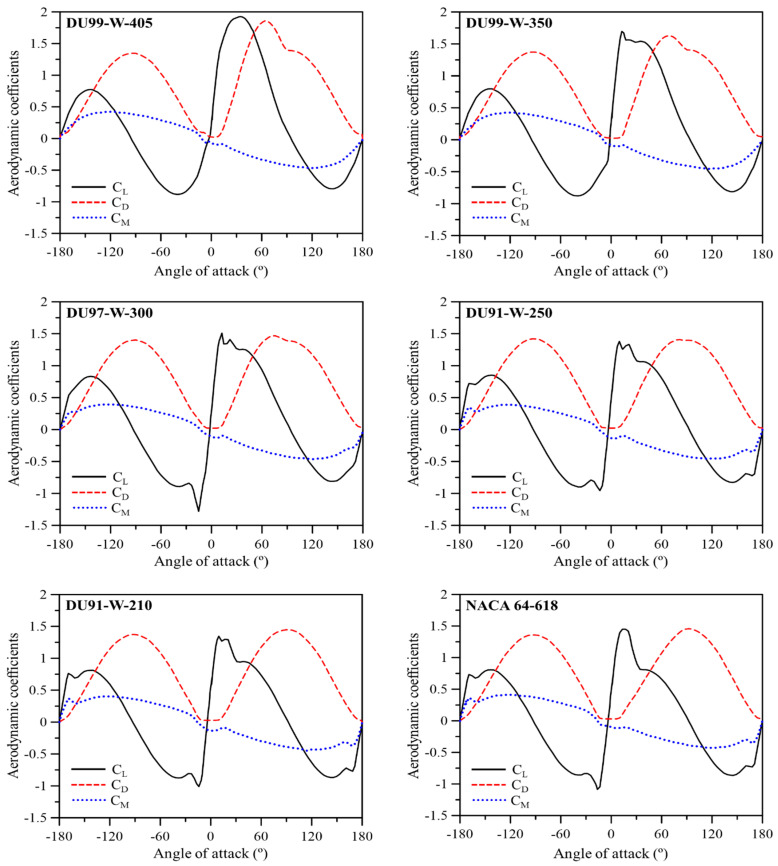
Aerodynamic coefficient of the airfoils in the SNL 61.5 m blade.

Subsequently, the angle of attack can be calculated using Equation (7). The calculated angle of attack is used to determine aerodynamic coefficients, such as the lift, drag, and pitching-moment coefficients. Figure 6 shows the lift coefficient $C_{L}$, drag coefficient $C_{D}$, and pitching-moment coefficient $C_{M}$ according to the angle of attack of each airfoil, which were experimentally obtained. $a_{0}$ and $a\prime_{0}$ are updated using the inflow angle and aerodynamic coefficients calculated above, as shown in Equation (8), and iterative calculations must be performed until the convergence condition presented in Equation (9) is met. In this study, the convergence condition ε was set to 0.001.

(7) α=φ−θ

(8) $$a=\frac{1}{\frac{4\sin^{2}\varphi }{\sigma (C_{L}\cos\varphi +C_{D}\sin\varphi )}+1},\;a\prime =\frac{1}{\frac{4\sin\varphi \cos\varphi }{\sigma (C_{L}\sin\varphi -C_{D}\cos\varphi )}-1}$$

(9) $$\left|a_{k}-a_{k-1}\right|<\varepsilon \;\mathrm{and}\;\left|a_{k}^{\prime }-a_{k-1}^{\prime }\right|<\varepsilon$$

where α is the angle of attack, θ is the twist angle of the airfoil, and σ=Bc/2πr holds. B is the number of blades in the wind turbine and c is the chord length.

**Figure 7:**
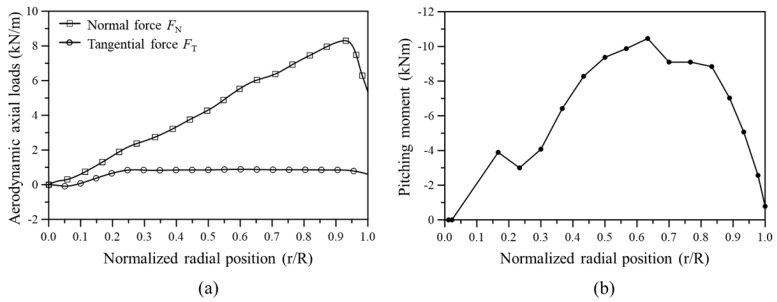
Distribution of aerodynamic loads along the blade span: (**a**) normal and tangential forces, (**b**) pitching moment.

Once the values of $a_{0}$ and $a\prime_{0}$ are finally determined, the lift, drag, and pitching moment acting on the aerodynamic center can be calculated using Equations (10)–(12). The lift and drag forces can be converted into normal and tangential forces through Equations (13) and (14). Figure 7 illustrates the distribution of the normal force, tangential force, and pitching moment calculated through the above processes.

(10) $$L=0.5\rho cV_{\mathrm{rel}}^{2}C_{L}dr$$

(11) $$D=0.5\rho cV_{\mathrm{rel}}^{2}C_{D}dr$$

(12) $$M=0.5\rho c^{2}V_{\mathrm{rel}}^{2}C_{M}dr$$

where ρ is the air density, and the relative velocity is calculated as $V_{\mathrm{rel}}=\sqrt{\;{\left[(1-a)V_{0}\right]}^{2}+{\left[(1+a\prime )\Omega r\right]}^{2}}$.

(13) $$F_{N}=L\sin\varphi -D\cos\varphi$$

(14) $$F_{T}=L\cos\varphi +D\sin\varphi$$

## 3. Results and Discussion

### 3.1. Validation of Finite Element Model

Before applying GPLRC to the wind turbine blade, it is necessary to verify the reliability of the developed analysis model through a comparison with the results presented in previous studies. Table 4 compares the weight of each material used in the analysis models of the present study and a previous study. The weight of each material and the total weight of the analysis model are similar to the values presented in the previous study [27].

Table 5 compares the natural frequencies of the SNL 61.5 m blade model obtained in this study and previous studies. Although the natural frequencies of the analysis models presented in Table 5 were different, the reliability of the analysis model and free vibration analysis of the present study has been verified in that the magnitudes of the natural frequencies were similar; furthermore, all of the observed mode shapes were the same as the mode order increased.

**Figure 8:**
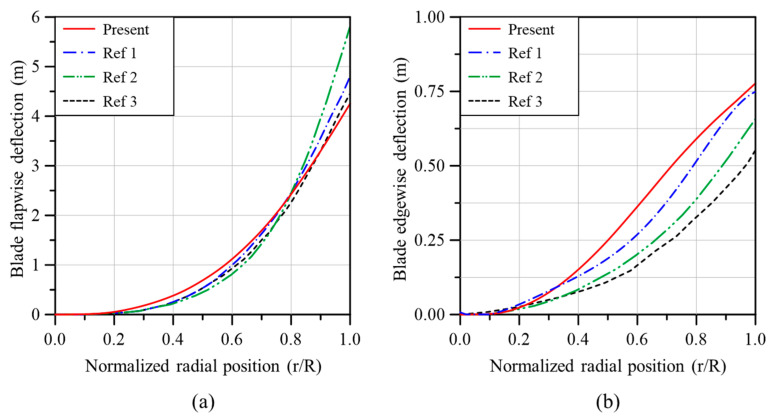
Comparisons of blade deflection due to aerodynamic loads: (**a**) flapwise direction, (**b**) edgewise direction (Ref 1: [31], Ref 2: [32], Ref 3: [33]).

Figure 8a,b compares the blade deflection in the flapwise and edgewise directions between the analysis models of the present study and previous studies. The deflection in the flapwise direction is quite similar to the results of previous studies, but the results presented in each study are different from each other for the deflection in the edgewise direction. The analysis model of the present study showed the largest deflection in the edgewise direction, but the maximum deflection that occurred at the blade tip was similar to the result of Ref. [31].

**Figure 9:**
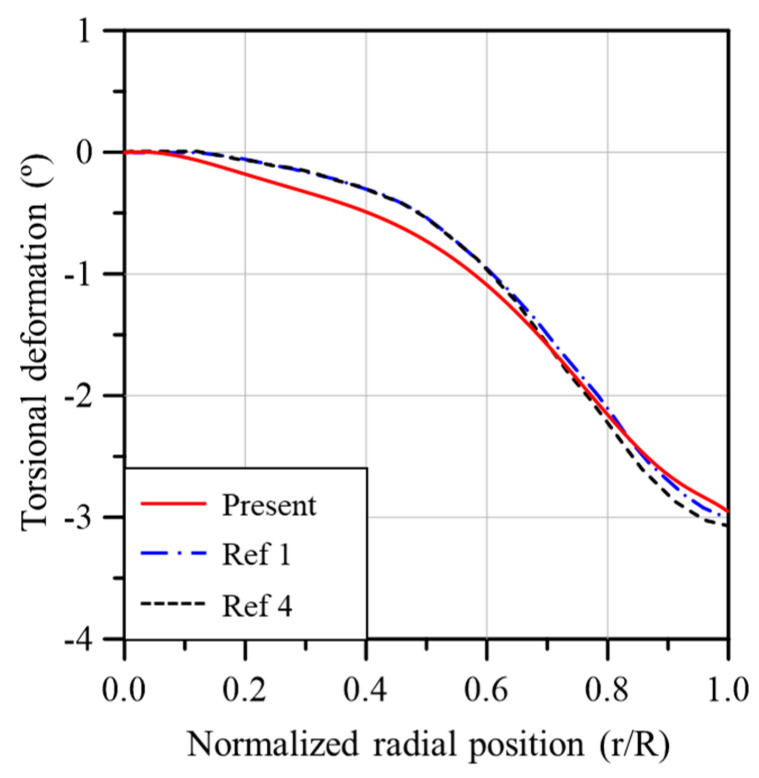
Comparisons of blade torsional deformation due to aerodynamic loads (Ref 1: [31], Ref 4: [34]).

Figure 9 compares the torsional deformation of the analysis models of the present study and previous studies. All the results are quite similar in terms of torsional deformation. Since the results in Figure 8 and Figure 9 are analysis results obtained using the aerodynamic loads calculated based on BEMT, the reliability of the aerodynamic loads calculated in this study has also been verified.

### 3.2. Application of GPLRC to Wind Turbine Blade

**Figure 10:**
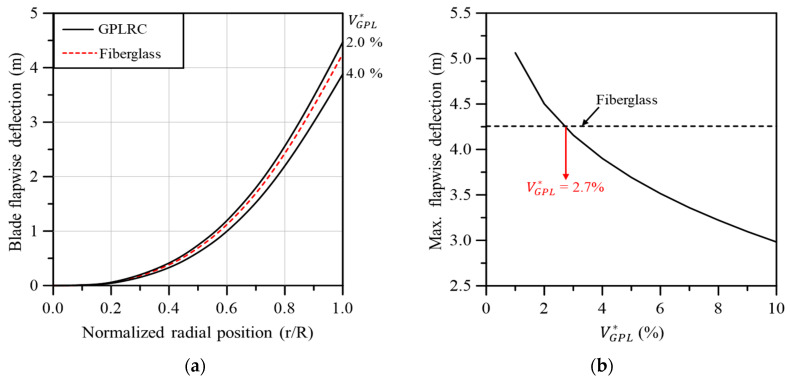
Flapwise blade deflection: (**a**) variation along the blade span for different volume fractions, (**b**) maximum values with respect to the GPL volume fraction.

In this section, the mechanical characteristics (e.g., deflection, torsion, and natural frequency) of wind turbine blades reinforced with GPLs are analyzed. The analysis model was created using GPLRC with the same thickness instead of E-LT-5500 (UD), Saertex (DB), and SNL (Triax), which are existing fiberglass composites. Finite element analysis was conducted while changing the volume fraction of GPL, $V_{CNT}^{*}$. Figure 10a compares the flapwise deflection of the GPL-reinforced wind turbine blade and the existing fiberglass composite wind turbine blade in the span direction. When GPL reinforcement was performed with $V_{GPL}^{*}$ between 2.0 and 4.0%, similar behavior to the flapwise deflection of the fiberglass composite-based wind turbine blade can be seen. The GPL content that exhibits similar behavior to the fiberglass composite-based wind turbine blade can be identified more precisely in Figure 10b. When $V_{GPL}^{*}$ is 2.7%, the maximum flapwise deflection that occurs at the blade tip is similar to that of the fiberglass composite-based blade.

**Figure 11:**
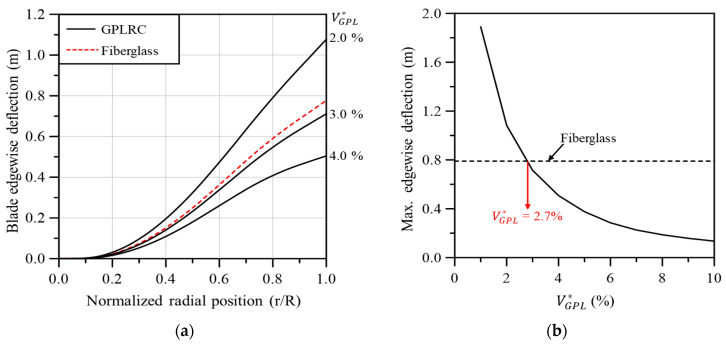
Edgewise blade deflection: (**a**) variation along the blade span for different volume fractions, (**b**) maximum values with respect to the GPL volume fraction.

Figure 11a compares the edgewise deflection of the GPL-reinforced wind turbine blade with that of the existing fiberglass composite-based blade in the span direction. Similar to flapwise deflection, the edgewise deflection is similar to that of the fiberglass composite-based blade when $V_{GPL}^{*}$ is between 2.0 and 3.0%. Compared to the flapwise deflection in Figure 10, the edgewise deflection seems to be quite sensitive to the change in $V_{GPL}^{*}$. This is due to the edgewise deflection being significantly smaller compared to the flapwise deflection, even though the changes in flapwise deflection and edgewise deflection caused by the change in $V_{GPL}^{*}$ are similar. Figure 11b shows the maximum edgewise deflection according to $V_{GPL}^{*}$. As with the flapwise deflection, the maximum edgewise deflection that occurs at the blade tip is similar to that of the fiberglass composite-based blade when $V_{GPL}^{*}$ is 2.7%.

**Figure 12:**
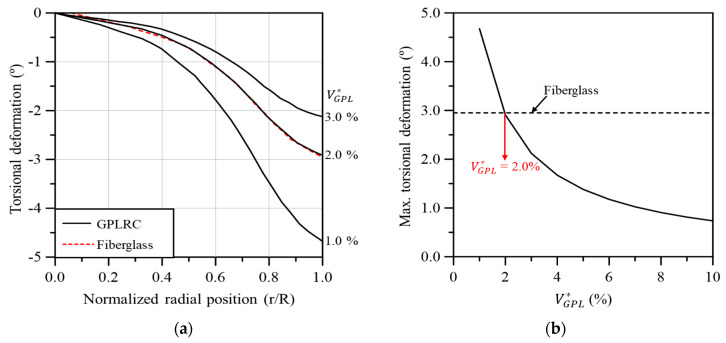
Torsional blade deformation: (**a**) variation along the blade span for different volume fractions, (**b**) maximum values with respect to the GPL volume fraction.

Figure 12a compares the torsional deformation of the GPL-reinforced wind turbine blade with that of the existing fiberglass composite-based blade in the span direction according to $V_{GPL}^{*}$. The torsional deformation of the GPL-reinforced wind turbine blade was quite similar to that of the fiberglass composite-based blade when $V_{GPL}^{*}$ was 2.0%. Figure 12b illustrates the maximum torsional deformation according to $V_{GPL}^{*}$. The maximum torsional deformation of the GPL-reinforced wind turbine blade is similar to that of the fiberglass composite-based blade when $V_{GPL}^{*}$ is 2.0%.

Based on the above results, the natural frequencies of the wind turbine blades were compared at $V_{GPL}^{*}=2.0\%$ and $V_{GPL}^{*}=2.7\%$, which exhibited mechanical characteristics similar to those of the existing fiberglass composite-based wind turbine blades. In addition, the weight difference of the wind turbine blade with GPLRC (ultra-light and high-strength nanomaterial) was also compared to analyze the degree of weight reduction compared to the use of the existing fiberglass composite. Table 6 shows the natural frequencies and weight of the fiberglass composite-based wind turbine blade and the GPL-reinforced wind turbine blade. The natural frequency of the GPLRC-based wind turbine blade was higher than that of the fiberglass composite-based wind turbine blade at all mode orders. The natural frequency increased as the volume fraction of GPL increased. This tendency is attributed to the relatively high stiffness and low mass of GPLRC, and it is in good agreement with the well-known natural frequency characteristics of GPL-reinforced composites. The weight of the GPLRC-based wind turbine blade was approximately 3620kg lower than that of the fiberglass composite-based blade, indicating that a weight reduction of more than 20% will be possible. Based on the results of this study, the application of GPLRC to wind turbine blades instead of existing fiberglass composites is expected to significantly reduce weight while maintaining strength that withstands aerodynamic loads at a similar level.

Table 7 compares the total masses and the estimated fabrication costs between fiberglass, CNTRC, and GPLRC. The fabrication costs for CNTs and GPLs were estimated by referring to the data provided by CTI Materials [35], where MWCNTs were chosen for CNTs. For the sake of conservative evaluation, industrial-grade (i.e., relatively lower cost) CNTs were selected, while research-grade (i.e., relatively higher cost) GPLs were chosen. The other fabrication cost was estimated based on the data provided by Bortolotti et al. [36]. The total masses of CNTRC and GPLRC blades are assumed to be the same based on the similar structural stiffness of CNTs and GPLs. From the table, it is found that the total fabrication cost of a CNTRC blade is 45.3% higher than one fiberglass blade. However, the total fabrication cost of a GPLRC blade is found to be only 5.7% higher than a fiberglass blade, even though the cost was assumed to be relatively higher. Thus, it has been justified that the total fabrication cost can be significantly reduced by replacing CNTs with GPLs, and the total weight of a wind blade can be remarkably reduced by replacing fiberglass with GPLs even though the total fabrication costs slightly increase.

## 4. Conclusions

In this study, the mechanical characteristics (e.g., deflection, torsion, and natural frequency) of the graphene platelet-reinforced composite (GPLRC) wind turbine blade were analyzed. The geometry and material properties of the finite element analysis model were modeled by referring to the SNL 61.5 m model, a 5 MW-class wind turbine blade model. The effective material properties of GPLRC were modeled using the Halpin–Tsai micromechanical model and the modified linear rule of mixture. Aerodynamic loads, the most crucial factors for the deflection and torsion of wind turbine blades, were calculated based on the blade element momentum theory (BEMT). The numerical analysis model was created using GPLRC with the same thickness instead of E-LT-5500 (UD), Saertex (DB), and SNL (Triax) materials. The applicability of GPLRC as future materials for wind turbine blades was examined through the numerical static bending, free vibration, and torsional stiffness. The numerical results draw the following main observations:

- A similar performance to the existing wind turbine blade was observed when $V_{GPL}^{*}$ was 2.7% for flapwise and edgewise deflection and 2.0% for torsional deformation.
- The natural frequency of the GPLRC-based wind turbine blade is higher than that of the existing fiberglass composite-based blade when $V_{GPL}^{*}$ is 2.0% and 2.7%.
- The production of 5 MW wind turbine blades using the materials discussed in this paper is expected to reduce weight by more than 20% while maintaining mechanical characteristics similar to those of existing blades.
- Reducing the weight of wind turbine blades is expected to significantly reduce the total construction cost of wind turbine support structures.
- The application of GPLRC remarkably reduces the fabrication cost of wind blades compared to other nanopillars such as CNT, and furthermore can also reduce the total weight of wind blades simultaneously.

These major observations justify that GPLRC has high potential as a cutting-edge material for the optimization of wind turbine blades.

## Figures and Tables

**Table 1:** Material properties of Epoxy and GPL [24].

Material	E (GPa)	$\nu_{12}$	ρ (kg/m^3^)
Epoxy	3.0	0.340	1200
GPL	1010.0	0.186	1060

**Table 2:** Examples of effective material properties of GPLRC.

$V_{GPL}$	$E_{eff}$ (GPa)	$\nu_{eff}$	$\rho_{eff}$ (kg/m^3^)
0.01 (1%)	11.8	0.338	1199
0.02 (2%)	20.7	0.337	1197
0.03 (3%)	30.0	0.335	1196
0.04 (4%)	38.5	0.334	1194

**Table 3:** Material properties of laminates [23].

Material	$E_{11}$ (GPa)	$E_{22}$ (GPa)	$G_{12}$ (GPa)	$\nu_{12}$	ρ (kg/m^3^)
Gelcoat	3.44	-	1.38	0.3	1235
E-LT-5500(UD)	41.8	14.0	2.63	0.28	1920
Saertex(DB)	13.6	13.3	11.8	0.49	1780
SNL(Triax)	27.7	13.65	7.2	0.39	1850
Foam	0.256	0.256	0.022	0.3	200
Carbon(UD)	114.5	8.39	5.99	0.27	1220

**Table 4:** Mass of materials used in the SNL 61.5 m wind turbine blade.

Model	Mass (kg)						
	Gelcoat	E-LT-5500 (UD)	Saertex (DB)	SNL (Triax)	Foam	Carbon (UD)	Total
Present	29	338	921	8726	4160	2655	16,829
Ref. [27]	29	376	916	8784	3953	2638	16,696

**Table 5:** Comparison of the natural frequencies of the SNL 61.5 m wind turbine blade.

Model	Natural Frequency (Hz)					
	1st Flapwise	1st Edgewise	2nd Flapwise	2nd Edgewise	3rd Flapwise	1st Torsion
Present	0.8415	0.9930	2.7269	3.5918	5.7255	6.7280
Ref. [23]	0.87	1.06	2.68	3.91	5.57	6.45
Ref. [28]	0.90	-	2.85	-	6.41	6.65
Ref. [29]	0.9194	1.0552	2.8106	3.8870	5.6904	6.7152
Ref. [30]	0.84	0.969	2.41	-	-	-

**Table 6:** Natural frequencies and weights of 5 MW wind turbine blades with fiberglass and GPLRC.

Material		Natural Frequency (Hz)						Weight (kg)
		1st Flapwise	1st Edgewise	2nd Flapwise	2nd Edgewise	3rd Flapwise	1st Torsion	
Fiberglass		0.8415	0.9930	2.7269	3.5918	5.7255	6.7280	16,829
GPLRC	$V_{GPL}^{*}=2.0\%$	0.8744	1.0352	2.9168	3.5834	6.1258	7.7406	13,216
	$V_{GPL}^{*}=2.7\%$	0.9412	1.1074	3.0376	3.9987	6.4612	8.6913	13,209

**Table 7:** Masses and fabrication costs of 5 MW wind turbine blades with fiberglass, CNTRC, and GPLRC [35,36].

Material		Material									Total
		Gelcoat	E-LT-5500(UD)	Saertex (DB)	SNL (Triax)	Foam	Carbon (UD)	Epoxy	MWCNT	GPL	
Cost per Mass (USD/kg)		7.23	1.87	3.00	2.86	7.23	30.00	3.63	450.00	90.00	
Mass (kg)	Fiberglass	29	338	921	8726	4160	2655	-	-		16,829
	CNTRC	29	-	-	-	4160	2655	6213	152	-	13,209
	GPLRC ($V_{GPL}^{*}=2.7\%$)	29	-	-	-	4160	2655	6213	-	152	13,209
Cost (USD)	Fiberglass	210	632	2763	24,956	30,077	79,650	-	-	-	138,288
	CNTRC	210	-	-	-	30,077	79,650	22,553	68,400	-	200,890
	GPLRC ($V_{GPL}^{*}=2.7\%$)	210	-	-	-	30,077	79,650	22,553	-	13,680	146,170

## Data Availability

The original contributions presented in the study are included in the article. Further inquiries can be directed to the corresponding author.

## Appendix A. Geometry Dimensions and Material Properties of Wind Blade

**Table A1:** Aerodynamic properties of the SNL 61.5 m wind turbine blade [25].

Blade Span (m)	Airfoil Type	Chord Length *L*_C_ (m)	Twist Angle (º)	Aero. Center *x*/*L*_C_
0	Circular	3.386	13.308	0.5
1.3667	Circular	3.386	13.308	0.5
10.25	DU99-W-405	4.557	13.308	0.275
14.35	DU99-W-350	4.652	11.480	0.275
22.55	DU97-W-300	4.249	9.011	0.275
26.65	DU91-W-250	4.007	7.795	0.275
30.75	DU91-W-250	3.748	6.544	0.275
34.85	DU91-W-210	3.502	5.361	0.275
38.95	DU91-W-210	3.256	4.188	0.275
43.05	NACA-64-618	3.010	3.125	0.275
47.15	NACA-64-618	2.764	2.319	0.275
51.25	NACA-64-618	2.518	1.526	0.275
54.6667	NACA-64-618	2.313	0.863	0.275
57.4	NACA-64-618	2.086	0.370	0.275
60.1333	NACA-64-618	1.419	0.106	0.275
61.5	NACA-64-618	1.086	0.000	0.275

**Table A2:** Stack IDs, names, and materials [25].

Stack ID	Stack Name	Material
1	Gelcoat	Gelcoat
2	Triax Skins	SNL(Triax)
3	Triax Root	SNL(Triax)
4	UD Carbon	Carbon(UD)
5	UD Glass TE	E-LT-5500(UD)
6	TE Foam	Foam
7	LE Foam	Foam
8	SW facesheet	Saertex(DB)
9	SW core	Foam

**Table A3:** Stacking sequence in each panel of the blade model along the span [25].

Blade Span (m)	LE	LE Panel	Spar Cap	TE	TE Reinforcement	TE Panel	Shear Web
0	1,2,3,2	1,2,3,2	1,2,3,2	1,2,3,2	1,2,3,2	1,2,3,2	-
1.3667	1,2,3,2	1,2,3,2	1,2,3,2	1,2,3,2	1,2,3,2	1,2,3,2	8,9,8
1.5	1,2,3,2	1,2,3,7,2	1,2,3,4,2	1,2,3,2	1,2,3,5,6,2	1,2,3,6,2	8,9,8
6.8333	1,2,3,2	1,2,3,7,2	1,2,3,4,2	1,2,3,2	1,2,3,5,6,2	1,2,3,6,2	8,9,8
9	1,2,2	1,2,7,2	1,2,4,2	1,2,2	1,2,5,6,2	1,2,6,2	8,9,8
43.05	1,2,2	1,2,7,2	1,2,4,2	1,2,2	1,2,5,6,2	1,2,6,2	8,9,8
45	1,2,2	1,2,7,2	1,2,4,2	1,2,2	-	1,2,6,2	8,9,8
61.5	1,2,2	1,2,2	1,2,2	1,2,2	-	1,2,2	8,9,8
